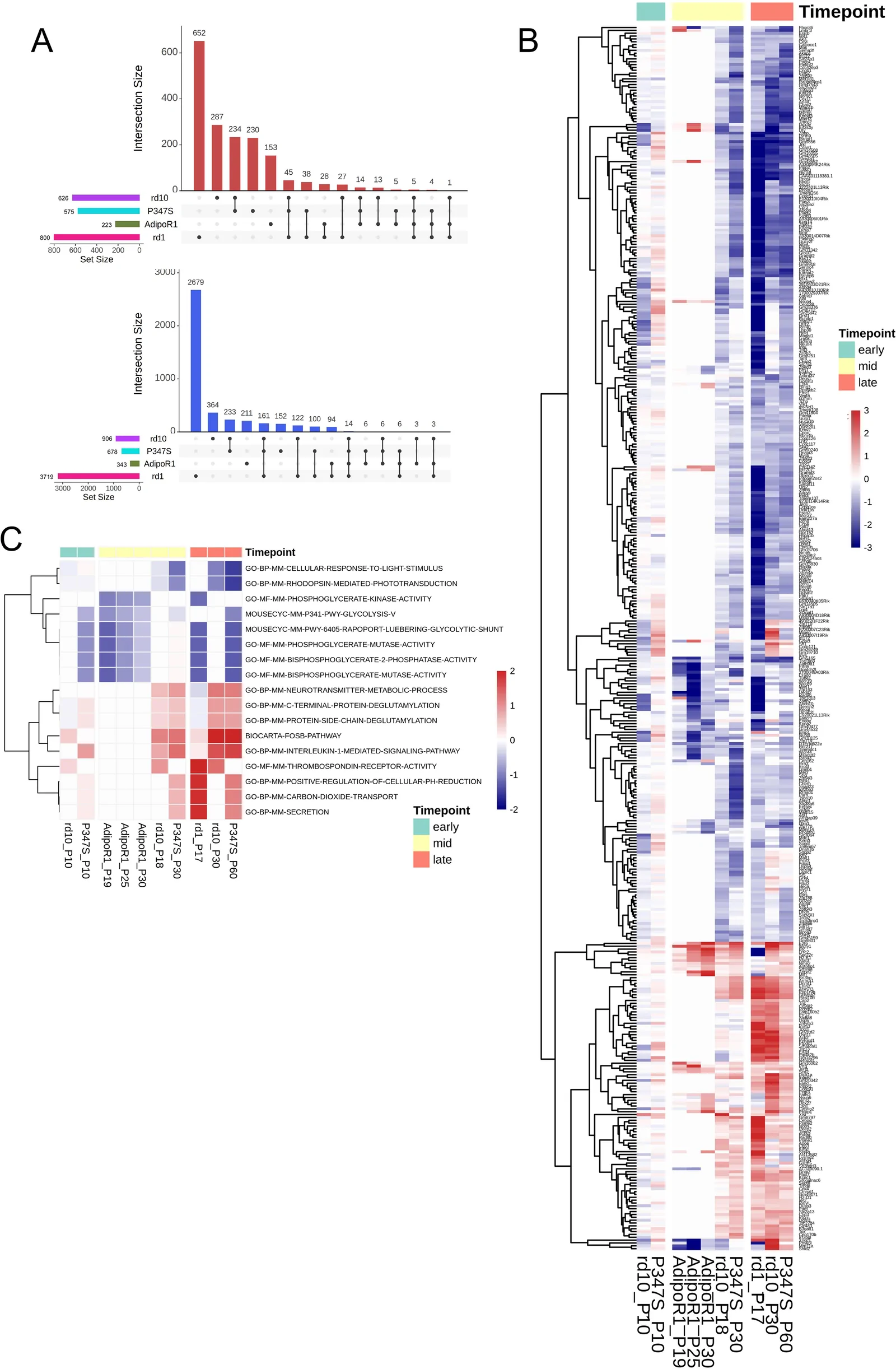# Single-cell transcriptome analysis highlights conserved transcriptional responses across multiple models of Retinitis Pigmentosa

**DOI:** 10.1016/j.gendis.2026.102241

**Published:** 2026-05-16

**Authors:** Santiago Negueruela, Martina Di Guida, Paola Brandi, Sandro Banfi

**Affiliations:** a Telethon Institute of Genetics and Medicine (TIGEM), Pozzuoli 80078, Italy; b Medical Genetics, Department of Precision Medicine, University of Campania “L. Vanvitelli”, Naples 80138, Italy; c Genomics and Experimental Medicine Program, Scuola Superiore Meridionale (SSM, School of Advanced Studies), Naples 80134, Italy

Retinitis pigmentosa (RP) is the most common form of inherited retinal dystrophy and is characterized by progressive rod degeneration followed by secondary cone loss (https://omim.org/entry/268000). Although numerous causative genes have been identified, it remains unclear to what extent different genetic forms of RP converge on shared pathogenic pathways.^1^ To address this issue, we performed single-cell transcriptomics across two murine RP models, rd10 and RHO-P347S (P347S), at three stages of photoreceptor degeneration, and validated shared signatures using publicly available datasets from *adiponectin receptor 1* (*AdipoR1*) knockout (*AdipoR1*^−/−^) and rd1 mice (Fig. 1A).

**Figure 1:**
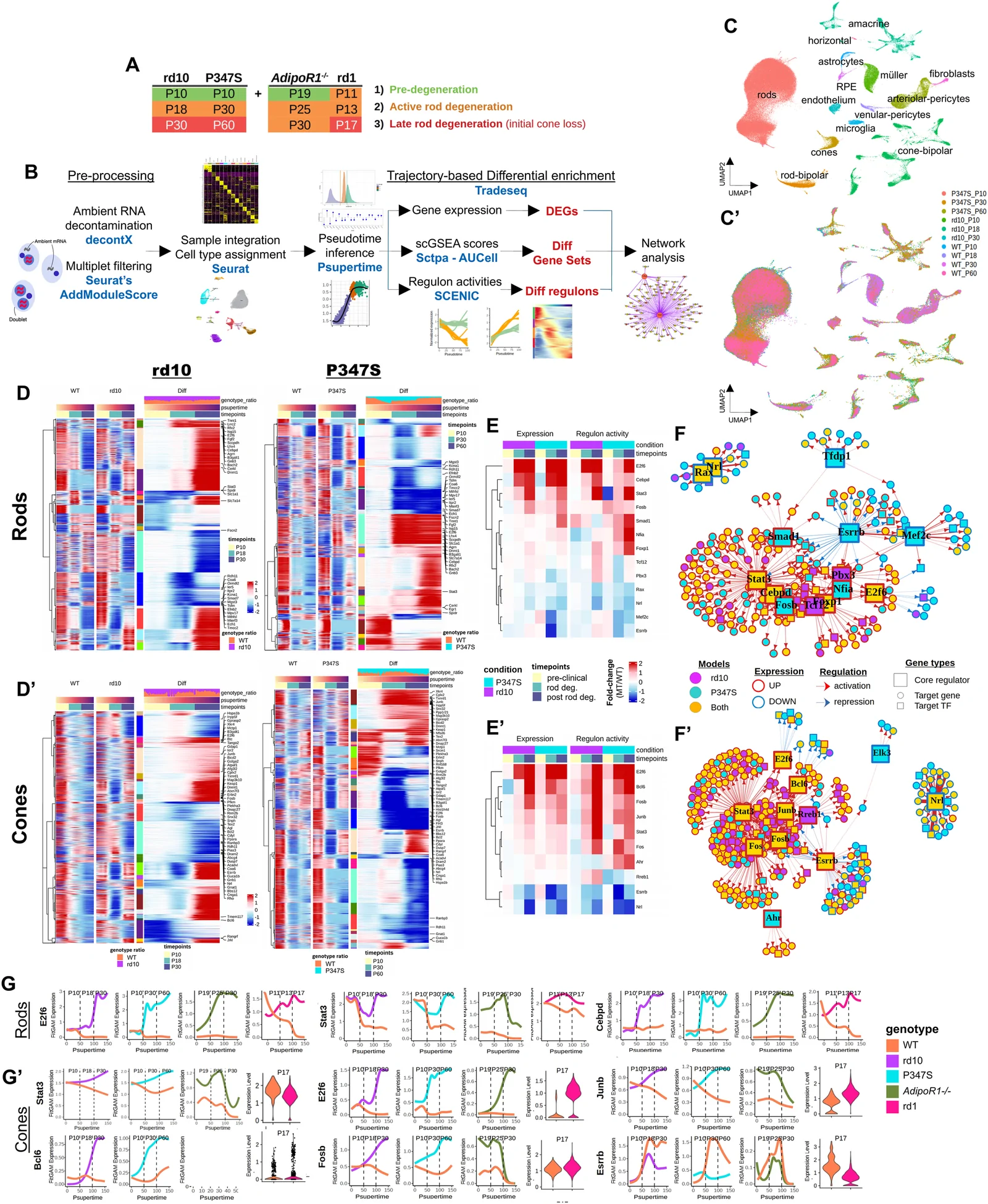
Photoreceptors from multiple retinitis pigmentosa (RP) models share transcriptomic signatures in response to retinal degeneration. **(A)** Time points selected (rd10 and P347S) or available (*AdipoR1*^−/−^ and rd1) for each RP model analyzed and the associated disease-development stage. **(B)** Schematic flowchart outlining the analytical pipeline used in this study, including single-cell RNA-sequencing data processing, differential expression analysis, and gene regulatory network (GRN) inference. **(C, C′)** UMAP visualization of single-cell retinal transcriptomes from rd10 and P347S mice, colored by (C) cell type identity or (C′) dataset (genotype and time point). **(D, D′)** Heatmaps illustrating pseudotemporal expression patterns of differentially expressed genes (DEGs) for rods (D) and cones (D′) from rd10 and P347S mice compared with wild-type (WT). For each model, the heatmaps on the left show expression levels for WT and mutant conditions, while the heatmap on the right displays the log expression ratio between mutant and WT conditions. Some DEGs of interest that were shared between models are highlighted. **(E, E′)** Heatmap showing fold-change in gene expression and AUCell regulon activity score inferred by pySCENIC for rods (E) and cones (E′) from rd10 and P347S models compared with WT mice. **(F, F′)** GRN for rods (F) and cones (F′) from rd10 and P347S mice, including differentially enriched regulons and DEGs. **(G, G′)** Expression profiles of selected differentially expressed transcription factors across rd10, P347S, *AdipoR1*^−/−^, and rd1 RP models in rods (G) and cones (G′). Note that for the rd1 model, it was possible to analyze only the last time point due to an insufficient number of cones in the earlier stages.

We generated an integrated single-cell RNA-sequencing atlas of wild-type, rd10, and P347S mouse retinas and ordered cells along pseudotime to distinguish degenerative changes from maturation processes (Fig. 1B–C'; Fig. S1). Three analytical layers were used to capture conserved disease signatures across the inferred trajectory: i) differentially expressed genes (DEGs), ii) AUCell-based pathway activity, and iii) SCENIC-derived regulon activity to identify transcription factors orchestrating the responses (Fig. 1B).

Because rods are the primary targets of RP, we first focused on their transcriptional responses. As expected, both rd10 and P347S displayed an increasing number of differentially expressed genes over pseudotime, peaking at late degeneration (Fig. 1D; Fig. S2A; Extended data 1). At this stage, the two models shared 54% up-regulated and 42% down-regulated genes. Key pro-survival factors, including *Stat3*, *Cebpd*, and *Fgf2*, displayed early induction. Pro-apoptotic genes, such as *Egr1* and *Apaf1*, alongside RP genes *Cerkl* and *Slc7a14*, were also up-regulated (Fig. S2B). Gene Ontology (GO) analysis indicated strong enrichment of programmed cell death, MAPK and JAK-STAT signaling, and immune-related pathways at the active degeneration stage (Fig. S2C–S2E; Extended data 2).

Cone responses were also examined, considering that only early degeneration events can be observed within the analyzed time window. Differentially expressed genes increased across pseudotime, with P347S showing more pronounced early changes than rd10 (Fig. 1D'; Fig. S3A; Extended dataset 3). Cones up-regulated neuroprotective genes shared with rods (*Cebpd*, *Fgf2*, *Bcl6*) and stress-induced transcriptional markers (*Fosb*, *Keap1*), while phototransduction genes such as *Guca1b* and *Gnb1* were down-regulated (Fig. S3B). Gene associated with synapsis, like *Cplx2* and *Lynx1*, were up-regulated. Enrichment analysis revealed progressive loss of cone phototransduction pathways and activation of IL-1 and CCR5 inflammatory signaling, stress-response programs, and neurotransmitter-related terms likely reflecting compensatory adaptation to rod loss (Fig. S3C–S3E; Extended data 4).

To identify transcription factors coordinating the above responses, we inferred regulon activities that exhibited robust correlation with corresponding transcription factor expression (Fig. 1E, E′). In rods, six regulons were consistently enriched in both models: *Stat3*, *Cebpd*, *E2f6*, and *Foxp1* were activated, while *Rax* and *Nrl* were down-regulated (Fig. 1E and F; Extended data 5). The involvement of *Stat3*, *Cebpd*, *Nrl*, and *Rax* aligns with their known roles in photoreceptor development and survival. Functional enrichment of target genes highlighted the association of *Stat3* and *Cebpd* for JAK/STAT, cytokine, FGF, insulin, and NGF signaling, while *E2f6* targets were linked to cell cycle regulation, DNA damage repair, and apoptosis, and *Foxp1* to GPCR signaling and synaptic regulation (Fig. S4A and S4B; Extended data 6).

In cones, six regulons were enriched across both models (*Bcl6*, *Fosb*, *Junb*, *Stat3*, and *Fos* up-regulated and *Nrl* down-regulated). Additionally, *Esrrb* and *E2f6* were robustly up-regulated at the transcript level (Fig. 1E', F'; Extended dataset 7). The Immediate early genes *Fosb*, *Junb*, and *Fos* represent core stress-responsive regulators, whereas *Bcl6* is associated with anti-apoptotic signaling. Functional enrichment analysis in regulon targets revealed shared processes with rods, including insulin and cytokine response and FGF signaling, supporting the presence of convergent survival pathways (Fig. S4C and S4D; Extended dataset 8). Metabolic enrichment (glucose import, glucose homeostasis, lipid metabolism) further suggests metabolic remodeling during cone stress.

To validate the transcriptional changes shared between the rd10 and P347S models, we decided to perform a meta-analysis comparing them with already available single-cell RNA-sequencing datasets from two additional RP murine models, namely *AdipoR1*^−/^^−^ (Fig. S5A–S5D) and rd1 (Fig. S5E–S5H). Each of these RP models is driven by distinct genetic mutations and pathophysiological mechanisms. Specifically, the RHO–P347S model features a class I rhodopsin mutation that causes protein mislocalization and subsequently proteostatic stress. The rd10 model (missense *Pde6b* mutation) impairs cGMP hydrolysis, leading to calcium overload and metabolic collapse, whereas the rd1 model (nonsense *Pde6b* mutation) completely abolishes PDE6B function, resulting in a drastically accelerated disease progression. Finally, the AdipoR1-knockout model exhibits disrupted lipid metabolism, compromising outer segment integrity. Overall, different types of cellular stress and rates of progression trigger specific responses. However, while convergent mechanisms may be fewer in number compared with model-specific responses (Fig. S6A and S7A), they are of high interest from a therapeutic standpoint.

The meta-analysis yielded both differentially expressed genes and enriched GO terms shared by most of the analyzed models. In rods, results indicate profound metabolic adaptations to elevated ATP demand, with up-regulated genes associated with mitochondrial boost and overload, including *Idh2* and *Slc25a37*. The up-regulation of *Pfkm*, *Pygm*, and *Slc2a3* suggests a compensatory shift toward aerobic glycolysis. The signaling machinery also adapts via decreased cAMP (*Adcy6* down-regulation; *Pde10a* up-regulation) and activated phospholipase C. This, alongside reduced Na^+^/K^+^-ATPase, increases cytosolic Ca^2+^, activating calpains. These proteases dismantle microtubules, which, together with protein deglutamylation and the compensation attempt by the AMAM pathway, suggest ciliary collapse (Fig. S6B and S6C). STAT3, a transcription factor identified in the gene regulatory network (GRN) analysis (Fig. 1E and F), seems to be at the center of these survival responses, either through the STAT3/EDN2/FGF2 axis or by interacting with CEBPD to modulate inflammation.

This study had some limitations for the cone meta-analysis regarding the two additional RP models. Only the third time point (P17) was included for rd1, as insufficient cone numbers were present at the previous stages. On the other hand, the last time point for *AdipoR1* knockout is still too early to observe cone degeneration. Taking that into account, we still managed to identify common mechanisms in cones (see methods). While rods attempt a compensatory glycolytic boost, cones show a terminal suppression of these pathways, probably due to the often-reported reduction in glucose uptake. Both photoreceptors exhibit increased protein deglutamylation, indicating a problem with the tubulin code also in cones. Phototransduction is down-regulated, probably to limit the energy demand and prioritize cell survival. We also found an increase in the CO_2_ transport and PH reduction, matching the previously described hyperoxia suffered by cones after rod death (Fig. S7B and S7C). Regarding the transcription factor identified in our GRN analysis (Fig. 1E'–F′), FosB signaling would be mediating a survival response together with JunB, up-regulated in every model. E2F6 was up-regulated in both photoreceptors, indicating a common mechanism to prevent cell cycle re-entry. *Stat3* showed strong up-regulation in most models and likely plays an increasingly prominent role as cone degeneration progresses.

This study provides the first comprehensive single-cell transcriptomic comparison of multiple RP models across distinct stages of degeneration. Our analyses reveal a robust set of conserved transcriptional and regulatory responses shared across genetically diverse models. Core transcription factors, particularly *Stat3*, *Cebpd*, and *E2f6*, emerge as central coordinators of stress, immune, and pro-survival programs in both rods and cones, suggesting that these factors represent mutation-independent molecular hubs of retinal degeneration in RP.

The pronounced activation of *Stat3* aligns with its dual role in the retina. Although STAT3 down-regulation is required for photoreceptor maturation and rhodopsin expression, its overactivation is a hallmark of retinal injury and degenerative disease.^2^ The consistent induction of *Cebpd* across models further underscores the importance of this stress-responsive transcription factor, which can interact with STAT3, promote cell survival, and perform immunomodulation in multiple contexts.^3^ *E2f6* is a transcriptional repressor that antagonizes pro-proliferative E2F family members and may act as a compensatory brake during degeneration. Since STAT3 can drive E2F-dependent cell-cycle programs,^4^ and aberrant cell-cycle re-entry is a known trigger of neuronal apoptosis,^5^ *E2f6* induction may help to limit cell death during chronic degeneration.

The recurrence of these transcription factors and pathways across four distinct RP models highlights their potential as targets for mutation-agnostic therapeutic intervention. Future work should clarify their mechanistic roles through chromatin immunoprecipitation-sequencing analyses in purified retinal cell types and ATAC-seq profiling to define dynamic changes in chromatin accessibility and transcription factor occupancy during degeneration. Integrating these approaches will help resolve direct regulatory interactions and refine the regulatory landscape of the photoreceptor responses in RP. While this study provides a promising computational blueprint, future experimental validation is essential. Specifically, functional perturbation studies or pharmacological experiments will be required to confirm the neuroprotective potential of these targets *in vivo*.

Expanding comparative analyses to additional RP models and later stages of cone degeneration will further strengthen the framework for mutation-independent therapeutic strategies, especially as cone preservation is crucial for maintaining vision in patients who are typically diagnosed after substantial rod loss. While our study emphasizes shared mechanisms to inform broadly applicable treatments, model-specific pathways will also be essential to define early pathogenic triggers and to develop complementary precision medicine approaches.

## CRediT authorship contribution statement

**Santiago Negueruela:** Writing – review & editing, Writing – original draft, Visualization, Software, Methodology, Investigation, Formal analysis, Conceptualization. **Martina Di Guida:** Writing – review & editing, Investigation. **Paola Brandi:** Writing – review & editing, Investigation. **Sandro Banfi:** Writing – review & editing, Writing – original draft, Supervision, Resources, Project administration, Funding acquisition, Conceptualization.

## Ethics declaration

All animal experiments were approved and conducted in accordance with the Institutional Ethics Committee at the TIGEM institute. Studies on mice were conducted following institutional guidelines for animal research (ARRIVE guidelines) and approved by the Italian Ministry of Health, Department of Public Health, Animal Health, Nutrition and Food Safety, in accordance with the law on animal experimentation (article 7; D.L. 116/92; protocol number: 254/2018-PR).

## Data availability

Single-cell RNA-sequencing datasets generated for the rd10 and P347S models are available in the GEO repository GSE296646. Samples for *AdipoR1*^−/−^ and rd1 mice can be found in the GEO repositories GSE183206 and GSE212183, respectively.

## Funding

This work was supported by the Italian Telethon Foundation (to S.B.), the European Union under the Next Generation EU program (No. PE00000006 CUP H93C22000660006-MNESYS to S.B.), and the Italian Ministry of University and Research under the EJP RD program (project Solve-RET, to S.B.) and under the Project PRIN2022 (No. 2022R9BKZN_002 to S.B.).

## Conflict of interests

The authors report no competing interests.

## Figures and Tables

**figs1:**
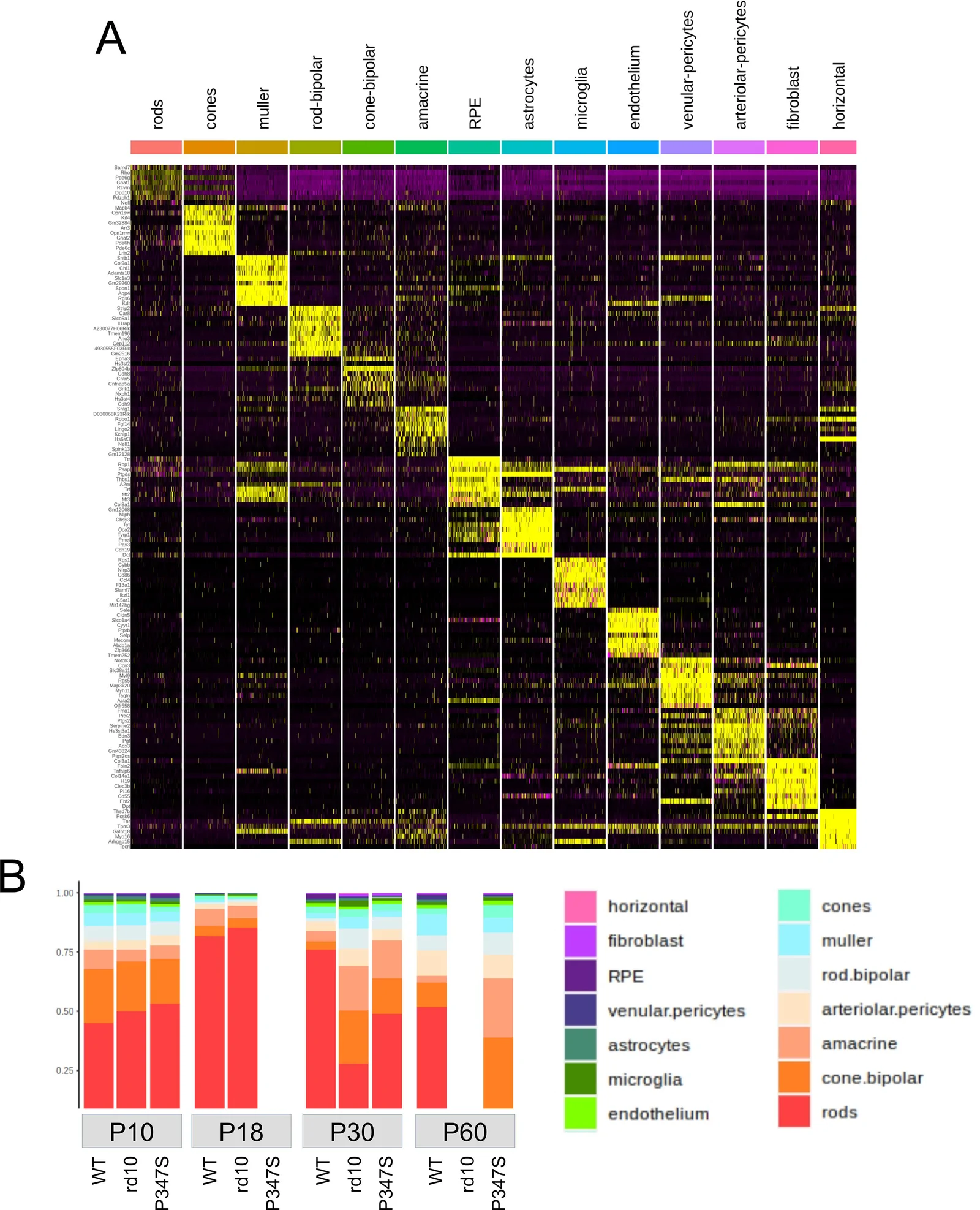


**figs2:**
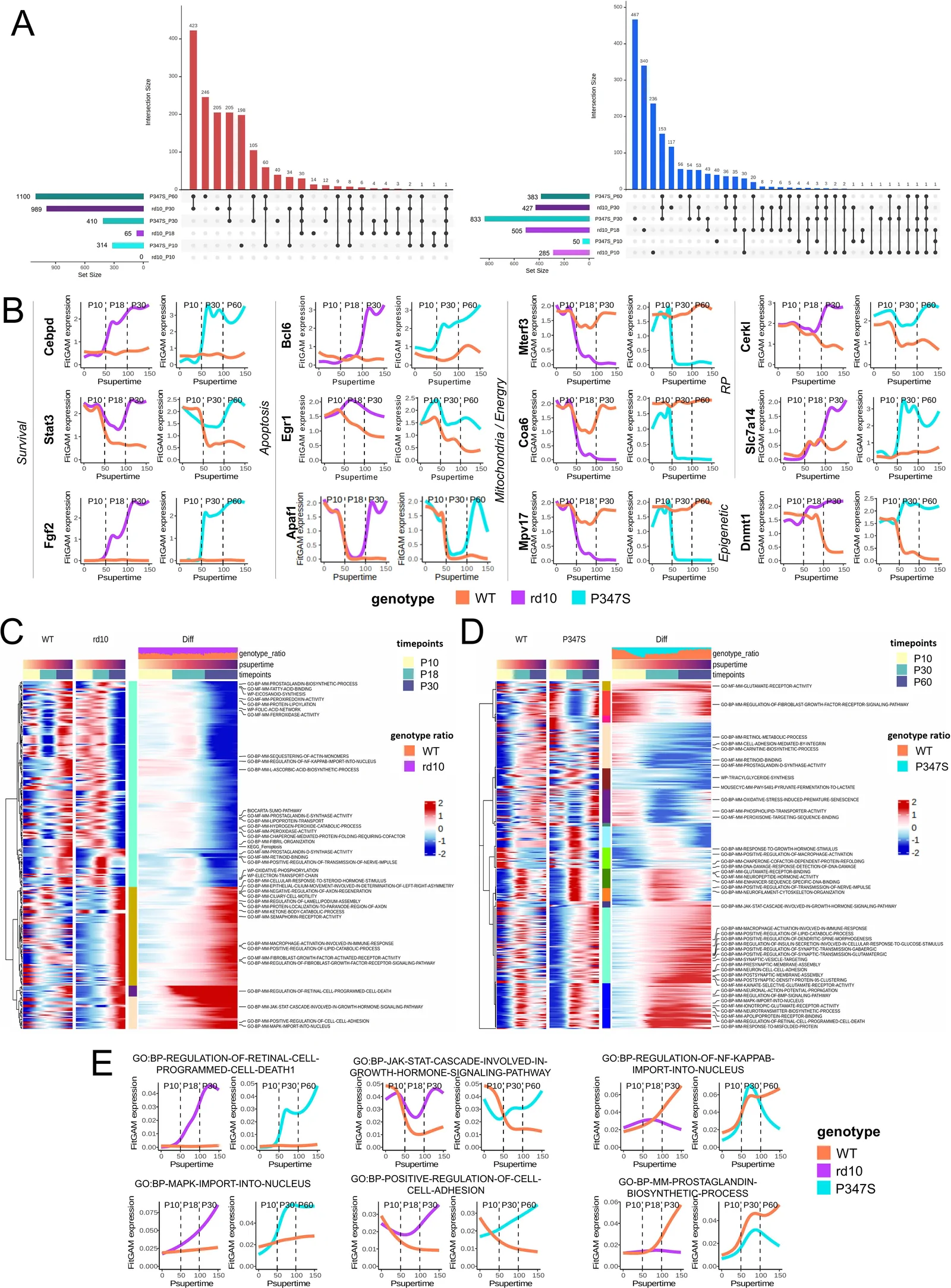


**figs3:**
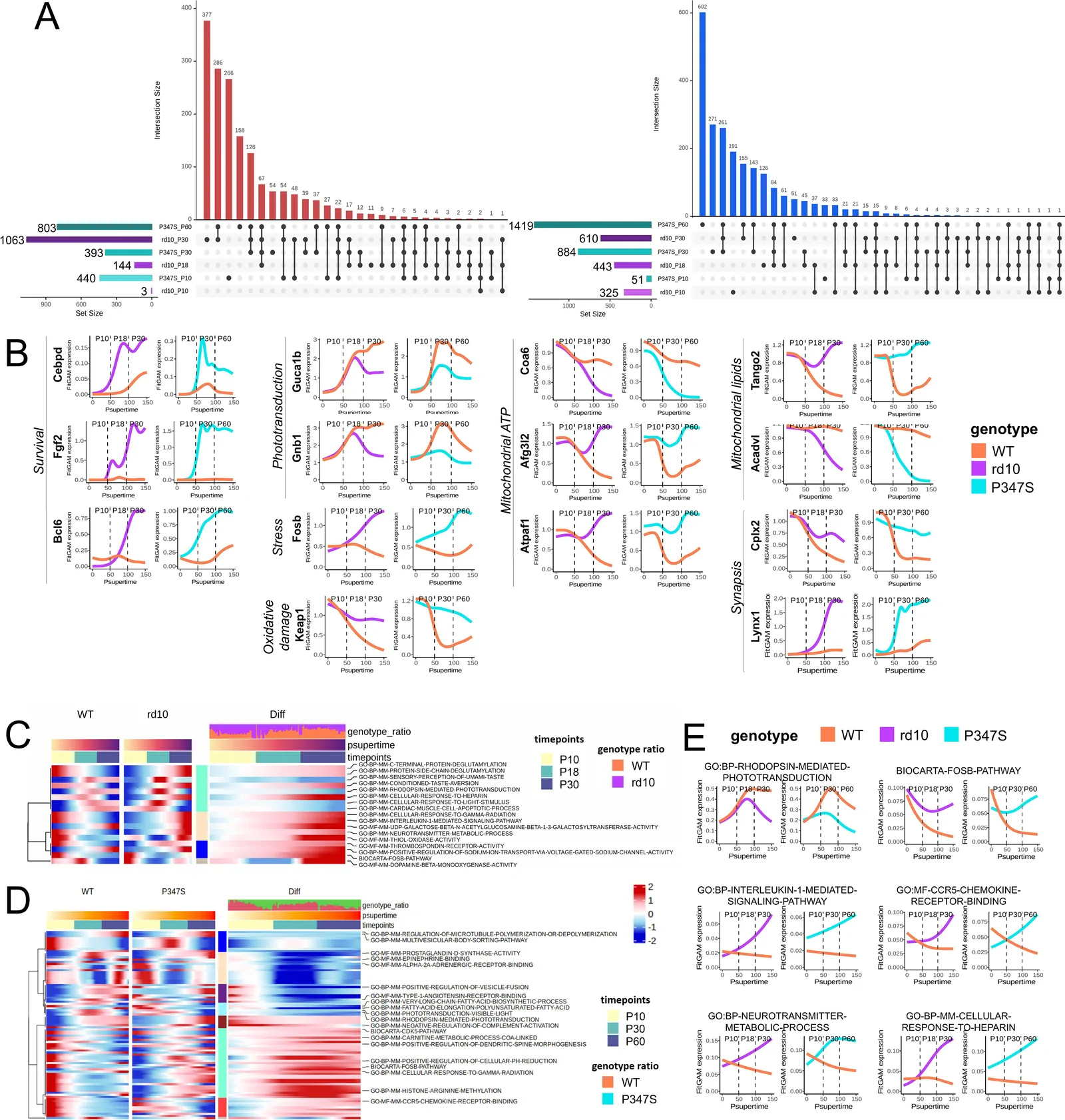


**figs4:**
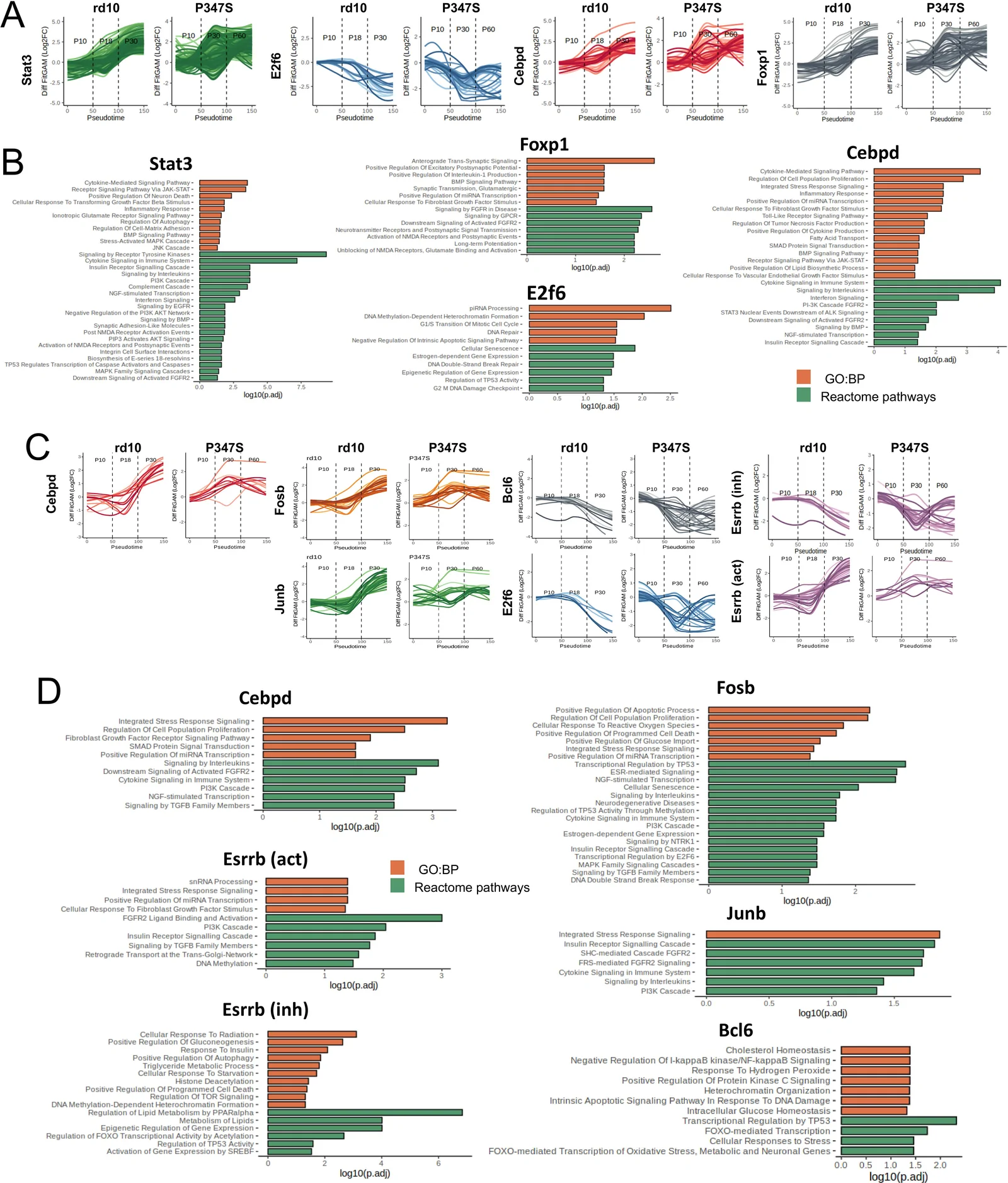


**figs5:**
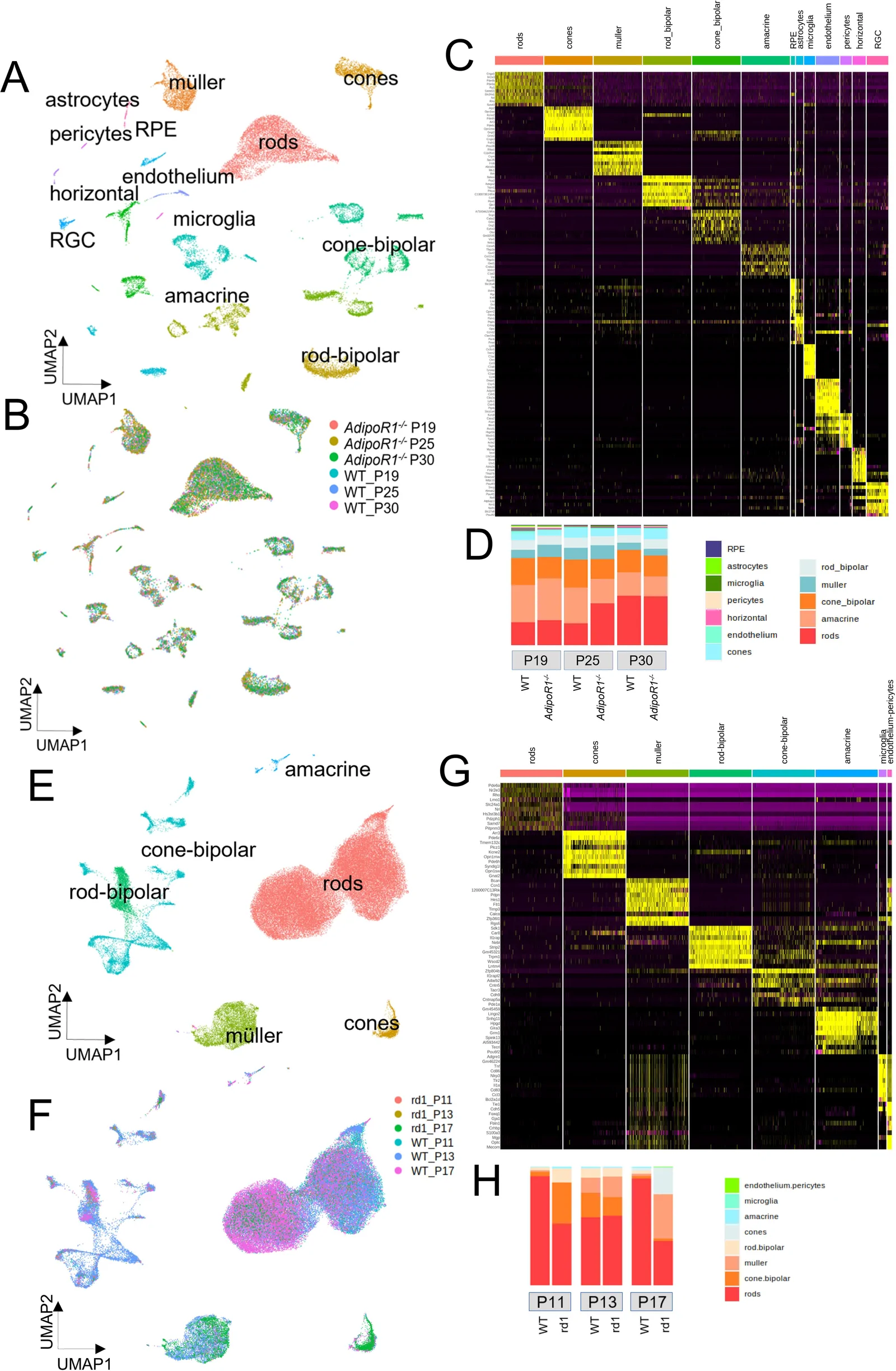


**figs6:**
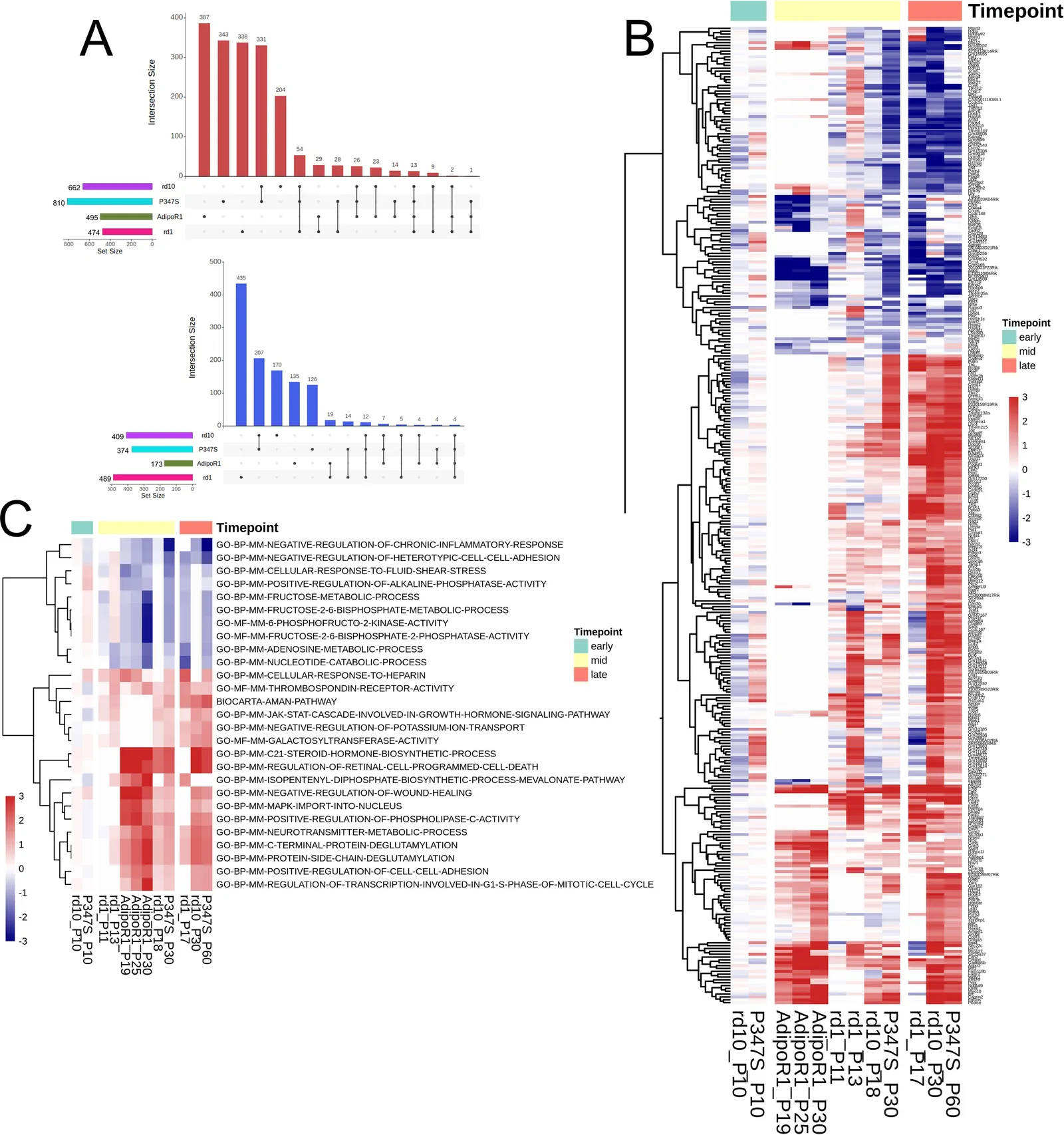


**figs7:**