# Supplementary material for: Single-cell transcriptome analysis highlights conserved transcriptional responses across multiple models of Retinitis Pigmentosa
Source: Genes Dis. 2026 May 16;14(3):102241. doi: 10.1016/j.gendis.2026.102241 (PMC13734888; doi:10.1016/j.gendis.2026.102241)
Supplement: Supplementary file 1 [file mmc1.docx]

**METHODS:**

**Murine models**

Studies on mice were conducted following institutional guidelines for animal research (ARRIVE guidelines) and approved by the Italian Ministry of Health (article 7; D.L. 116/92; protocol number: 254/2018‐PR) and the Institutional Ethics Committee at TIGEM (Pozzuoli, Italy). Mice were housed under SPF‐like conditions in ventilated cages (maximum four per cage), with controlled light/dark cycles, temperature, and humidity, and with food and water provided ad libitum. Both sexes were included. Feeding and sanitary conditions were monitored by a committee that included veterinarians.

RHO-P347S mice were generated by crossing P347S+/+ transgenic (A1 line) with C57BL/6J mice, producing P347S+/− offspring. The presence of the rd8 allele was excluded. The A1 line was obtained from Dr. Jane G. Farrar’s laboratory^1^ and maintained by homozygous crosses. rd10 mice were obtained from Dr. Mathias Seeliger’s group^2^. Pups were produced by crossing heterozygous Pde6brd10/+ mice, generating both wild-type and homozygous mutant animals within the same litter.

**Single Cell Transcriptomics:**

scRNA-seq samples for AdipoR1-/- mice were obtained from Lewandowski et al.^3^, and those for rd1 mice were obtained from Chen et al.^4^

***- Dissection and dissociation of retinas into single cells***

Retinas were dissected in the CO_2_-independent medium (Gibco, cat. # 18045088) and dissociated into single cells using the Worthington Papain Dissociation System (Worthington Biochemical Corporation, cat. # LK003150) followed by two DPBS washes (ThermoFisher, cat. # 14190144) and centrifuging at 1,200 xg for 5 minutes. Single-cell suspensions from three individual retinas were pooled per sample, resuspended in PBS containing 0.04% BSA, and filtered through 40 μm cell strainers (Biologix, Cat. #15-1040). Cell concentration was determined using a Bio-Rad TC20 Automated Cell Counter (Bio-Rad, Cat. #1450102) and adjusted to 1,000 cells/μl.

***- Preparation of scRNA-seq library and sequencing***

scRNA-seq was conducted using the NEGEDIA NGD0050R platform (Negedia s.r.l). The cell suspension was loaded onto the Chromium Single Cell G Chip Kit (10x Genomics) and processed using the Chromium Single Cell Controller (10x Genomics) to generate single-cell gel bead emulsions following the manufacturer’s protocol. For library preparation, cDNA synthesis and final libraries were prepared using the Single Cell 3′ Library and Gel Bead Kit V3.1 (10x Genomics). cDNA quality was assessed using the High Sensitivity D5000 ScreenTape on the Agilent 4200 TapeStation system (Agilent Technologies) and High Sensitivity DNA D1000 ScreenTape (Agilent Technologies). Libraries were then sequenced on the Illumina NovaSeq 6000.

***- scRNA-seq Data Processing***

Reads were aligned to the mm10 reference genome (refdata version 2020-A) with Cell Ranger v7.0.0^5^ with default mapping parameters and including introns. Ambient RNA contamination was corrected using decontX v1.14.2^6^. Most ambient RNA is originated from rods, so their expression was conserved. Multiple detection was based on co-expression of marker genes. Cell type identity scores were calculated for each droplet using Seurat’s^7^ AddModuleScore function with a curated panel of cell type-specific genes (Supplementary Table 3). Events associated with multiple cell types (hybrid transcriptome) were considered multiplets and excluded. Cells that passed quality control were further processed Datasets were integrated with Seurat v5^8^ using the CCA algorithm. Dimensionality reduction was performed with PCA, followed by KNN graph construction (30 PCs), clustering (resolution = 1.2), and UMAP visualization. Cell types were identified based on the same module scores used in multiplet filtering.

***- Trajectory-based differential expression***

To perform differential expression analyses over time, we employed a comprehensive pipeline combining gene expression imputation, pseudotime modeling, and statistical testing. Gene expression was imputed with ALRA^9^, improving detection of lowly expressed transcripts. Pseudotime trajectories were inferred with psupertime v0.2.6^10^. Pseudotime values were scaled to ensure each time point represented the same range to facilitate visualization.

Trajectory-based differential expression analysis was carried out using tradeSeq v1.12.0^11^. fitGAM function was used to fit a negative binomial distribution with 9 knots for each gene across the psupertime trajectory. Differential expression was assessed using the conditionTest function, applying a log2FC threshold of 1. Genes were retained if they passed in at least one condition per time point expression (expr), frequency (pct) and logFC thresholds. Parameters were adapted for rods (min._pct 20%, min._expr 0.5, min_logFC 0.5) and cones (min. pct 20%, min._expr 0.15, min._logFC 0.5). For the cones from the rd1 RP model, DE analysis was limited to P17 using MAST in Seurat with the following filters: min_padj 0.05, min_pct 0.10, min_logFC 1.

DEGs were clustered with clusterExperiment Bioconductor package and visualized as heatmaps with ComplexHeatmap. For comparisons between models, expression differences were calculated as log2 ratios of mutant vs wild-type fitGAM-smoothed expression. Expression was scaled per row only for condition-specific heatmaps to highlight relative changes.

***- Gene Ontology Enrichment Analysis***

GO enrichment was assessed using both single-time point and trajectory-based approaches. Single-time point GSEA was conducted using the fgsea Bioconductor package v1.24.0^12^ with gene sets from MSigDB, while ORA was performed via enrichR v3.3 R package^13^ with a defined background list. For trajectory-based GSEA, AUCell scores were computed using AUCell^14^ implemented through testScTPA v1.0 R package^15^. Differentially enriched GO terms were identified analyzing AUCell scores along the pseudotime trajectory using tradeSeq following the same approach applied to gene expression. GO terms were considered significantly enriched if they met the following criteria: logFC > 0.75, pct > 0.15, and mean AUCell score > 0.0025.

In the rd1 there are not enough cone cells for the first and second timepoints to perform the trajectory analysis with tradeSeq. Thus, a cross-sectional differential analysis was performed at T3 using the limma package^16^. To maintain consistency with the log-link nature of the GAMs used in tradeSeq, AUCell scores were log2-transformed (with a pseudo-count of 0.01) prior to linear modeling. A linear model was fitted to the transformed scores:

$$Y_{pathway}=\beta_{0}+\beta_{1}\cdot\text{Condition}+ \epsilon$$

**Y_GO_term_**: Observed data to be modeled, it represents the log2-transformed AUCell scores for a specific GO term in a single cell.

**ϐ_0_**: The intercept, the expected value of Y when the predictor variable is zero. In this case, the average pathway activity score for WT cells at T3.

**Condition**: The predictor denotes which experimental group a given cell belongs to.

**ϐ_1_**: The coefficient/slope represents the change in Y when the condition changes from WT(0) to MT(1).

**ϵ**: The residuals capture the variance in pathway activity that the model cannot explain by the cell’s condition.

**- Gene Regulatory Network Analysis**

GRNs were constructed using the SCENIC framework^14^: (1) Co-expression modules were determined with GENIE3^17^ implemented via pySCENIC’s docker container (docker://aertslab/pyscenic:0.12.1), subsampling up to 1,000 cells per sample and cell type. (2) Motif enrichment analysis with CisTarget prune co-expression modules into regulons. (3) Regulon activity per cell is computed using AUCell (auc_threshold = 0.01). To refine the network, TF-target relationships were retained only if importance score exceeded 0.0025 and protein-protein interaction (PPI) combined score from STRING v12^18^ was greater than 200. Differentially enriched regulons were assessed using AUCell scores along the pseudotime trajectories as input for tradeSeq. Regulons were considered significantly enriched if they met all of the following criteria in at least one condition: logFC > 0.75, pct > 0.20, mean AUCell score > 0.005, and pvalue < 0.1.

REFERENCES:

1.	Loscher CJ, Hokamp K, Kenna PF, et al. Altered retinal microRNA expression profile in a mouse model of retinitis pigmentosa. *Genome Biol*. 2007;8(11):R248. doi:10.1186/gb-2007-8-11-r248

2.	Hüttl S, Michalakis S, Seeliger M, et al. Impaired channel targeting and retinal degeneration in mice lacking the cyclic nucleotide-gated channel subunit CNGB1. *J Neurosci*. 2005;25(1):130-138. doi:10.1523/JNEUROSCI.3764-04.2005

3.	Lewandowski D, Foik AT, Smidak R, et al. Inhibition of ceramide accumulation in AdipoR1-/- mice increases photoreceptor survival and improves vision. *JCI Insight*. 2022;7(4). doi:10.1172/jci.insight.156301

4.	Chen Y, Dong Y, Yan J, et al. Single-Cell Transcriptomic Profiling in Inherited Retinal Degeneration Reveals Distinct Metabolic Pathways in Rod and Cone Photoreceptors. *Int J Mol Sci*. 2022;23(20). doi:10.3390/ijms232012170

5.	Zheng GXY, Terry JM, Belgrader P, et al. Massively parallel digital transcriptional profiling of single cells. *Nat Commun*. 2017;8:14049. doi:10.1038/ncomms14049

6.	Yang S, Corbett SE, Koga Y, et al. Decontamination of ambient RNA in single-cell RNA-seq with DecontX. *Genome Biol*. 2020;21(1):57. doi:10.1186/s13059-020-1950-6

7.	Stuart T, Butler A, Hoffman P, et al. Comprehensive Integration of Single-Cell Data. *Cell*. 2019;177(7):1888-1902.e21. doi:10.1016/j.cell.2019.05.031

8.	Hao Y, Stuart T, Kowalski MH, et al. Dictionary learning for integrative, multimodal and scalable single-cell analysis. *Nat Biotechnol*. 2024;42(2):293-304. doi:10.1038/s41587-023-01767-y

9.	Linderman GC, Zhao J, Roulis M, et al. Zero-preserving imputation of single-cell RNA-seq data. *Nat Commun*. 2022;13(1):192. doi:10.1038/s41467-021-27729-z

10.	Macnair W, Gupta R, Claassen M. psupertime: supervised pseudotime analysis for time-series single-cell RNA-seq data. *Bioinformatics*. 2022;38(Suppl 1):i290-i298. doi:10.1093/bioinformatics/btac227

11.	Van den Berge K, Roux de Bézieux H, Street K, et al. Trajectory-based differential expression analysis for single-cell sequencing data. *Nat Commun*. 2020;11(1):1201. doi:10.1038/s41467-020-14766-3

12.	Korotkevich G, Sukhov V, Budin N, Shpak B, Artyomov MN, Sergushichev A. Fast gene set enrichment analysis. Preprint posted online June 20, 2016. doi:10.1101/060012

13.	Kuleshov M V, Jones MR, Rouillard AD, et al. Enrichr: a comprehensive gene set enrichment analysis web server 2016 update. *Nucleic Acids Res*. 2016;44(W1):W90-7. doi:10.1093/nar/gkw377

14.	Aibar S, González-Blas CB, Moerman T, et al. SCENIC: single-cell regulatory network inference and clustering. *Nat Methods*. 2017;14(11):1083-1086. doi:10.1038/nmeth.4463

15.	Zhang Y, Zhang Y, Hu J, et al. scTPA: a web tool for single-cell transcriptome analysis of pathway activation signatures. *Bioinformatics*. 2020;36(14):4217-4219. doi:10.1093/bioinformatics/btaa532

16.	Ritchie ME, Phipson B, Wu D, et al. limma powers differential expression analyses for RNA-sequencing and microarray studies. *Nucleic Acids Res*. 2015;43(7):e47. doi:10.1093/nar/gkv007

17.	Huynh-Thu VA, Irrthum A, Wehenkel L, Geurts P. Inferring regulatory networks from expression data using tree-based methods. *PLoS One*. 2010;5(9). doi:10.1371/journal.pone.0012776

18.	Szklarczyk D, Kirsch R, Koutrouli M, et al. The STRING database in 2023: protein-protein association networks and functional enrichment analyses for any sequenced genome of interest. *Nucleic Acids Res*. 2023;51(D1):D638-D646. doi:10.1093/nar/gkac1000

**Figure S1** Cell type annotation of single-cell transcriptomes for rd10 and P347S retinas. **(A)** Heatmap displaying the 14 cell clusters identified based on the expression of representative marker genes. **(B)** Bar plot showing the proportional distribution of cell types identified in Figure 1C across genotypes and time points.

**Figure S2** Rods from the rd10 and P347S models share a significant number of differentially expressed genes (DEGs) and enriched Gene Ontology (GO) terms. **(A)** Upset plots displaying the overlap of up-regulated (left, red bars) and down-regulated (right, blue bars) DEGs across rd10 and P347S samples. **(B)** Expression profiles for selected DEGs common to both rd10 and P347S models, grouped according to functional categories. **(C, D)** Heatmaps depicting pseudotemporal patterns of AUCell scores for enriched GO terms in rods from rd10 (C) and P347S (D) mice compared with wild-type (WT). The heatmaps on the left of each panel show AUCell scores for WT and mutant conditions, while the heatmap on the right indicates the log AUCell ratio between mutant and WT conditions. Some differentially enriched terms of interest that were shared between models are highlighted. **(E)** AUCell scores for selected enriched GO terms.

**Figure S3** Shared transcriptional changes in cones from rd10 and P347S mice. **(A)** Upset plots for up-regulated (left, red bars) and down-regulated (right, blue bars) differentially expressed genes (DEGs) in cones from rd10 and P347S mice. **(B)** Expression profiles for selected DEGs common to both rd10 and P347S mice, grouped according to their biological roles. **(C, D)** Heatmaps displaying pseudotemporal patterns of AUCell scores for enriched Gene Ontology (GO) terms in cones from rd10 (A) and P347S (B) mice compared with wild-type (WT). The heatmaps on the left of each panel show AUCell scores for WT and mutant conditions, while the heatmap on the right indicates the log AUCell ratio between mutant and WT conditions. Some differentially enriched terms of interest that were shared between models are highlighted. **(E)** AUCell scores for selected enriched GO terms across the pseudotime trajectory.

**Figure S4** Predicted function for shared differentially enriched regulons in photoreceptors from rd10 and P347S mice. **(A)** Expression profiles of differentially expressed genes (DEGs) belonging to each regulon in rods from rd10 and P347S mice. **(B)** Over-representation analysis (ORA) enrichment analysis of DEGs corresponding to each regulon in (A) for BP (Biological Processes) and RP (Reactome Pathways) in rods. **(C)** Expression profiles of DEGs belonging to each regulon in cones from rd10 and P347S mice. **(D)** ORA enrichment analysis of DEGs corresponding to each regulon in Figure S4A for BP and RP in cones.

**Figure S5** Single-cell transcriptomes from *AdipoR1*^–/–^ and rd1 mice. **(A, B, E, F)** UMAP visualization of single cells from *AdipoR1*^–/–^ (A, B) or rd1 (E, F) retinas, colored by (A, E) cell type identity or (B, F) dataset (genotype and time point). **(C, G)** Heatmap displaying the 13 clusters based on the expression of representative marker genes for *AdipoR1*^–/–^ (C) or rd1 (G) mice. **(D, H)** Bar plot showing the proportion of cell types identified in (A or E), separated by genotype and time point in *AdipoR1*^–/–^ (D) or rd1 (H) retinas.

**Figure S6** Meta-analysis in rods comparing rd10, P347S, *AdipoR1*^–/–^, and rd1 retinitis pigmentosa (RP) models. **(A)** Upset plot for up-regulated (left, red bars) and down-regulated (right, blue bars) differentially expressed genes (DEGs) in rods. **(B, C)** Heatmaps displaying DEGs (B) or enriched Gene Ontology (GO) terms (C) shared by most of the RP models analyzed.

**Figure S7** Meta-analysis in cones comparing rd10, P347S, *AdipoR1*^–/–^, and rd1 retinitis pigmentosa (RP) models. **(A)** Upset plot for up-regulated (left, red bars) and down-regulated (right, blue bars) differentially expressed genes (DEGs) in cones. **(B, C)** Heatmaps displaying DEGs (B) or enriched Gene Ontology (GO) terms (C) shared by most of the RP models analyzed.
