# Supplementary material for: Single-cell transcriptome analysis highlights conserved transcriptional responses across multiple models of Retinitis Pigmentosa
Source: Genes Dis. 2026 May 16;14(3):102241. doi: 10.1016/j.gendis.2026.102241 (PMC13734888; doi:10.1016/j.gendis.2026.102241)
Supplement: Supplementary file 2 [file mmc10.docx]

**Supplementary Table 1. List of differentially expressed genes in rods shared across the RP models analyzed**

**UPREGULATED:**

| 4 models | Antxr2, Cebpd, Cog7, E2f6, Fgf2, Gadd45b, Gnb3, Limch1, Mlf1, Nin, Pbxip1, Prtg, Stat3 |
| --- | --- |
| 3/4 models | 2010315B03Rik, 9330159F19Rik, 9330159M07Rik, Actr3b, Afg3l2, Agtpbp1, Atpaf1, B3galt1, Bach2, Bcl6, Btc, Caprin2, Ccdc177, Ccm2, Cend1, Cerkl, Cpsf3, Ctnnal1, Cux2, Dnmt1, Dpp10, Fancl, Fbxl7, Fgf2os, Flot2, Fosb, Gm12592, Gm14211, Gpr162, Hacd1, Hap1, Hook2, Ikzf4, Inpp5f, Itga4, Klhl29, Klhl8, Kremen1, Lonrf1, Lrmda, Mrps27, Myo10, Myo5b, Nav1, Ndufaf6, Nelfb, Nmnat3, Nr4a1, Nt5dc3, Pcolce, Pde10a, Pdlim3, Pid1, Pigu, Pkp2, Poglut1, Polr3h, Ptprn, Pygm, Qsox1, R3hcc1l, Rexo4, Rfx2, Rnf165, Scube1, Sec22c, Sept6, Sgcb, Skap2, Slc1a1, Slc2a13, Slc2a3, Snph, Spaca6, Stat2, Tnnt1, Traf3ip2, Twsg1, Vstm2b, Wnk3, Yars Zbtb7c, Zfyve28 |

**DOWNREGULATED:**

| 4 models | Aqp1, E130310I04Rik, Gm5165, Kpna2 |
| --- | --- |
| 3/4 models | 3010001F23Rik, Atad2, BC065403, Ccn4, Gm14508, Gm47543, Gm5617, Gzmm, Hist1h1c, Jrkl, Llgl2, Paqr4, Pias3, Plagl2, Ranbp6, Rpp25l, Spry2, Suv39h2, Tmem35a |
