# Supplementary material for: Single-cell transcriptome analysis highlights conserved transcriptional responses across multiple models of Retinitis Pigmentosa
Source: Genes Dis. 2026 May 16;14(3):102241. doi: 10.1016/j.gendis.2026.102241 (PMC13734888; doi:10.1016/j.gendis.2026.102241)
Supplement: Supplementary file 3 [file mmc11.docx]

**Supplementary Table 2. List of differentially expressed genes in cones shared across the RP models analyzed**

**UPREGULATED:**

| 4 models | Caprin2, E2f6, Ier2, Irf2bpl, Junb |
| --- | --- |
| 3/4 models | AC149090.1, Acp2, Afg3l2, Agtpbp1, Antxr2, Armcx1, Atxn7l3, B3galt1, Bri3bp, Btc, Cep170b, Clip3, Dipk1a, Dnmt1, Dusp3, Erlin2, Fads2, Fancl, Fbxl7, Fgf2, Gm20342, Gnai2, Gng5, Gprasp2, Gtf2ird2, H2-D1, H2-K1, Inpp5f, Itga4, Kcnc1, Kif5c, Lrrc2, Ncdn, Ogfrl1, Pam, Pbxip1, Pcgf3, Poteg, Ppp1r9b, Prtg, Ptpdc1, Ptprn, Pyroxd1, Rad50, Ralyl, Rasl10b, Reep2, Rock2, Scd2, Slc2a13, Slc4a3, Smarcal1, Smim4, St6galnac6, Stat3, Timp2, Tnc, Tnnt1, Tox, Trhde, Tsc22d4, Ttc13, Usp11, Wdfy1 |

**DOWNREGULATED:**

| 4 models | Cdyl, Gm11342, Gm44532, Gm5165, Igf2bp3, Igsf9, Kpna2, Plekhf2, Ranbp6, Serinc4, Tnfaip3, Traf3ip3, Trpc1, Wdr89 |
| --- | --- |
| 3/4 models | 1700029J07Rik, 2700049A03Rik, 3222401L13Rik, 3300002I08Rik, 9230114K14Rik, A330094K24Rik, A430010J10Rik, A930004D18Rik, A930006I01Rik, A930014D07Rik, Abca13, Abcg4, Acvr2b, Adcy6, Agtrap, Ak3, Akap1, Ap3d1, Arl4d, Avil, Bbof1, Bbs1, Bbs12, Bhlhe41, C920021L13Rik, CAAA01118383.1, Calcoco1, Ccdc117, Ccdc51, Ccdc9, Ccn4, Ccpg1os, Cdan1, Cdc42ep3, Cdr2, Cep89, Chac2, Cherp, Cngb1, Cox11, D1Ertd622e, Degs1, Dgcr8, Dusp7, E130310I04Rik, Eepd1, Eif2s3y, Endov, Epb41l4aos, Ero1l, Esrrb, Fam227a, Fbxo36, Fscn2, Glb1l2, Gm14505, Gm14508, Gm16055, Gm41804, Gm47543, Gm48505, Gm48552, Gm50240, Gm9856, Gm9918, Gpr152, Gpsm2, Gramd2, Gramd3, Guca1b, Gucy2f, Gulp1, Haus8, Hexdc, Hirip3, Hspa1b, Ift122, Jag1, Jrkl, Katnal2, Kbtbd3, Kcnj14, Klhl28, Llgl2, Lmbr1l, Lmnb1, Lpin1, Lrit3, Lrp4, Lyrm9, Mecr, Mfap1b, Mid1ip1, Mok, Morn1, Ms4a14, mt-Nd3, Mterf3, Mycbp, Mylip, Nelfb, Nlgn2, Nomo1, Nrm, Nt5e, Nubpl, Nxf1, Pank4, Paqr4, Pde6a, Pde6b, Pdia5, Peli3, Pias3, Pitpnm2os2, Plagl2, Plekha2, Plpp2, Polg2, Ppara, Ppm1n, Ppp1r42, Ptges2, Rab43, Rangrf, Rapgef4os1, Rbm4b, Rdh11, Rdh8, Reep6, Rhbdl3, Ric1, Riok1, Rncr4, Rrnad1, Sall2, Samd11, Sema3f, Setd4, Ska2, Skida1, Slc17a1, Slc24a1, Slc25a22, Slc26a2, Slco4a1, Slx1b, Snap23, Snhg6, Srp54a, St3gal1, Stk19, Styx, Susd3, Suv39h2, Syne3, Terf2, Timmdc1, Tmcc2, Tmem107, Tmem266, Tmem67, Ttll7, Ubxn11, Uckl1os, Uty, Vax2os, Vegfa, Wdr27, Zbtb6, Zfp523, Zfp597, Zfp617, Zfp692, Zfp770, Zpbp |
