# Supplementary material for: Single-cell transcriptome analysis highlights conserved transcriptional responses across multiple models of Retinitis Pigmentosa
Source: Genes Dis. 2026 May 16;14(3):102241. doi: 10.1016/j.gendis.2026.102241 (PMC13734888; doi:10.1016/j.gendis.2026.102241)
Supplement: Supplementary file 4 [file mmc12.docx]

**Supplementary Table 3. List of gene markers used to identify retinal cell types**

**Celltype:	Markers:**

rods	Pdc, Rcvrn, Sag, Rho, Gnat1

cones	Pde6h, Opn1sw, Gnat2, Pde6c, Arr3

bipolar1	Zfp804b, Car10, Gabrb3

bipolar2	Prkca, Pcp2

bipolar3	Nckap5, Kcnd3, Cntn6, Prkcb, Grik2

endothelium	Cldn5,	Ptprb, Flt1

pericytes1	Serpine2, Igfbp5, Igfbp4

pericytes2	Col1a2, Col3a1, Igfbp5

pericytes3	Rgs5, Myl9

amacrine1	Dab1,	Hs6st3

amacrine2	Snhg11, Lingo2, Sntg1

amacrine3	Dlgap1, Frmd5

astrocytes	Dct, Pmel, Tyrp1, Mlana, Mgll

muller	Crym, Dbi, Slc1a3, Rlbp1, Clu

RPE	Ttr, Rgr, Rdh5

microglia	Tyrobp, Fcer1g, C1qb, C1qa, C1qc, Ctss, Cd52

redcells	Hba-a2, Hbb-bt

horizontal	Onecut1, Adgrb1, Lhx1

RGC	Sncg, Nefl

cornea	Ifi202b, S100a6, S100a11, Sfn, Emp1
